# Cooperation between p21 and Akt is required for p53‐dependent cellular senescence

**DOI:** 10.1111/acel.12639

**Published:** 2017-07-09

**Authors:** Young Yeon Kim, Hye Jin Jee, Jee‐Hyun Um, Young Mi Kim, Sun Sik Bae, Jeanho Yun

**Affiliations:** ^1^ Peripheral Neuropathy Research Center College of Medicine Dong‐A University Busan 49201 Korea; ^2^ Department of Biochemistry College of Medicine Dong‐A University Busan 49201 Korea; ^3^ Department of Pharmacology School of Medicine Pusan National University Yangsan‐si 602‐739 Korea

**Keywords:** Akt, NOX4, p53, reactive oxygen species, senescence

## Abstract

Cellular senescence has been implicated in normal aging, tissue homeostasis, and tumor suppression. Although p53 has been shown to be a central mediator of cellular senescence, the signaling pathway by which it induces senescence remains incompletely understood. In this study, we have shown that both Akt and p21 are required to induce cellular senescence in response to p53 expression. In a p53‐induced senescence model, we found that Akt activation was essential for inducing a cellular senescence phenotype. Surprisingly, Akt inhibition did not abolish p53‐induced cell cycle arrest, but it suppressed the increase in intracellular reactive oxygen species (ROS) levels. The results of the cell cycle and morphological analysis suggest that p53 induced quiescence, not senescence, following Akt inhibition. Conversely, the inhibition of p21 induction abolished cell cycle arrest but did not affect the p53‐induced increase in ROS levels. Additionally, p21 and Akt separately controlled cell cycle arrest and ROS levels, respectively, during H‐Ras‐induced senescence in human normal fibroblasts. The mechanistic analysis revealed that Akt increased ROS levels through NOX4 induction, and increased Akt‐dependent NF‐κB binding to the NOX4 promoter is responsible for NOX4 induction upon p53 expression. We further showed that Akt activation upon p53 expression is mediated by mammalian target of rapamycin complex 2. In addition, p53‐mediated IL6 and IL8 induction was abrogated by Akt inhibition, suggesting that Akt activation is also required for the senescence‐associated secretory phenotype. Collectively, these results suggest that p53 simultaneously controls multiple pathways to induce cellular senescence through p21 and Akt.

## Introduction

Cellular senescence was first described by Hayflick *et al*. as the limited proliferative capacity of normal human fibroblasts. While this phenomenon, termed ‘replicative senescence’, is known to contribute to organismal aging processes, another type of cellular senescence, termed ‘premature senescence’ or ‘stress‐induced senescence’, is considered a barrier to tumorigenesis because it acts to remove precancerous cells (Collado *et al*., 2005; Collado & Serrano, 2010). Cellular senescence has also been recently implicated in various pathophysiological conditions, such as wound healing and tissue fibrosis (Munoz‐Espin & Serrano, 2014; Tominaga, 2015). Given the importance of cellular senescence in tissue homeostasis and various diseases, it is important to understand the underlying regulatory pathways.

Extensive previous studies have shed light on the essential role of the tumor suppressor p53 in the induction and maintenance of cellular senescence (Kuilman *et al*., 2010; Rufini *et al*., 2013). With regard to replicative senescence, p53 activation by the DNA damage signaling cascade in response to telomere erosion is considered a critical step in the induction of cellular senescence (Kuilman *et al*., 2010). p53 has also been shown to play pivotal roles in various premature senescence models, such as anticancer drug‐ and H‐Ras oncogene‐induced senescence (OIS) (Ferbeyre *et al*., 2002).

Although increasing evidence indicates that p53 expression is sufficient to induce cellular senescence (Sugrue *et al*., 1997; Rufini *et al*., 2013), the downstream targets of p53 in the induction of senescence still need to be identified. Several p53 downstream targets, including PAI‐1, PML, miR‐34 and p21, have been shown to accumulate in senescent cells and contribute to p53‐induced senescence (Qian & Chen, 2013). PML and miR‐34 promote p53 function and premature senescence through a positive feedback loop (Yamakuchi & Lowenstein, 2009; Qian & Chen, 2013). It has also been reported that ectopic expression of PAI‐1 is sufficient to induce the senescence phenotype in normal fibroblasts (Kortlever *et al*., 2006).

p21/CDKN1A is a well‐known p53 target gene that has been shown to play a critical role during the induction of p53‐dependent cellular senescence by inducing cell cycle arrest, mainly during the G1/S‐phase (Brugarolas *et al*., 1995). However, it is unlikely that cell cycle arrest is solely responsible for the various phenotypic changes that provoke cellular senescence, and the p21‐independent induction of senescence has also been reported (Wyllie *et al*., 2003). Reactive oxygen species (ROS) are also considered important determinants or mediators of both cellular senescence and organismal aging (Lu & Finkel, 2008). A significant body of evidence indicates that an increase in intracellular ROS levels often occurs during various types of cellular senescence that contributes to senescence induction (reviewed in Balaban *et al*. (2005); Lu & Finkel (2008)). The findings of a previous report demonstrating an increase in ROS levels during p53‐induced cellular senescence, as well as the inhibition of senescence induction following treatment with an antioxidant, *N*‐acetylcysteine, further support the notion that ROS play an important role in p53‐induced senescence (Jung *et al*., 2004). However, the molecular pathway by which p53 expression increases ROS levels is not fully understood.

In this study, we have found that Akt is critical to p53‐induced increases in intracellular ROS levels. Importantly, our results have revealed that the cell cycle arrest and increased ROS levels are separately regulated by p21 and Akt, respectively. Our results have further suggested that cooperation between the functions of p21 and Akt is essential for inducing p53‐dependent premature cellular senescence.

## Results

### Akt is activated in p53‐induced senescence

**Figure 1:**
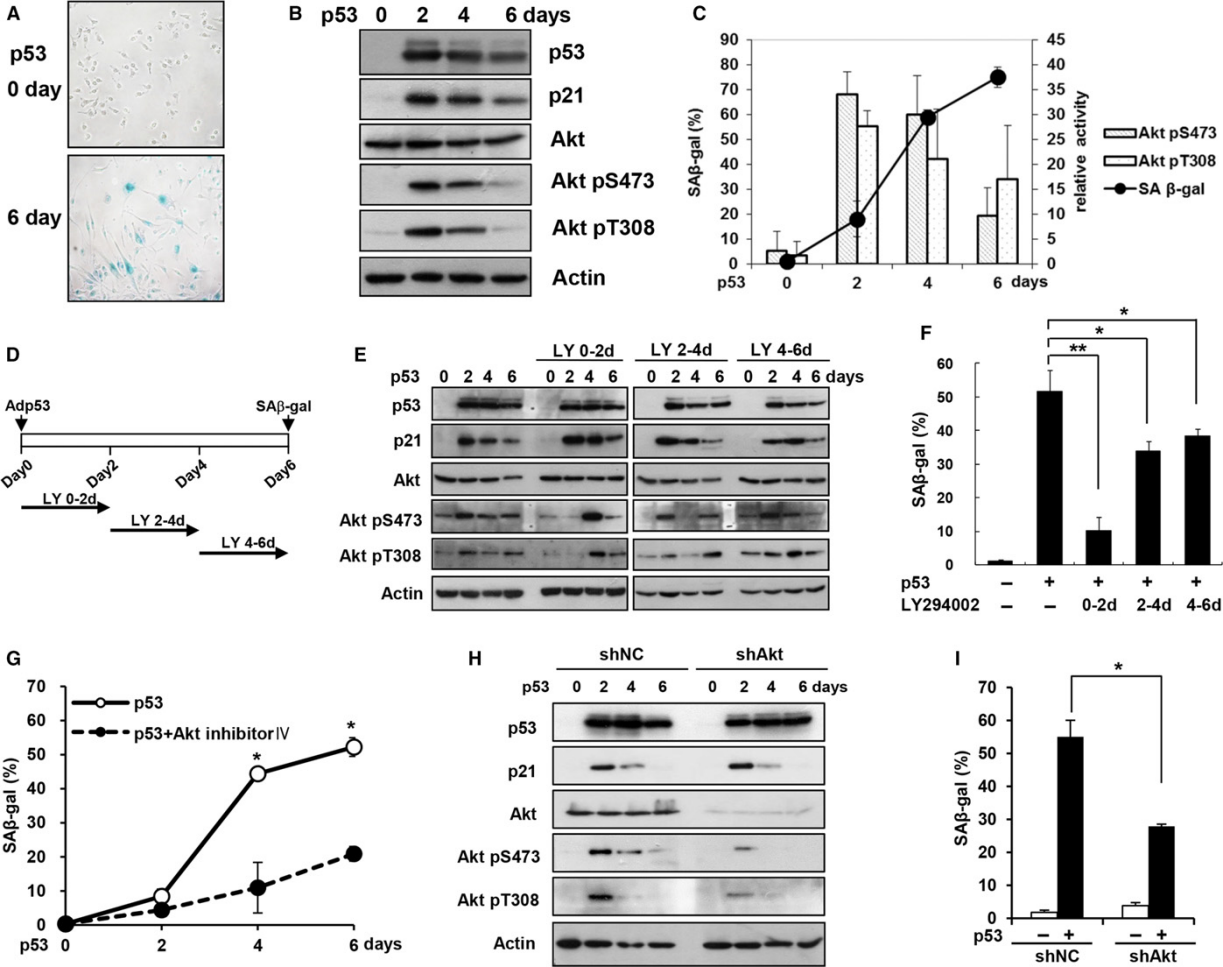
Akt activation is required for p53‐induced senescence. (A–C) EJ cells were infected with a p53 adenovirus at 100 MOI. SA‐β‐gal staining was examined after 6 days, and a representative image is shown (A). EJ cells were harvested at the indicated time points, and cell lysates were subjected to Western blot analysis using the indicated antibodies (B). The levels of Akt pS473 and pT308 from three replicate experiments were calculated and plotted, along with the percentage of SA‐β‐gal‐positive cells (C). (D–F) EJ cells were infected with a p53 adenovirus and treated with LY294002 (20 μm) for the indicated durations. The schematic diagram depicts the LY294002 treatment procedure (LY) (D). EJ cells were harvested at the indicated time points and subjected to Western blot analysis using the indicated antibodies (E). SA‐β‐gal staining was performed after 6 days (F). (G) EJ cells were treated with the Akt inhibitor IV (1.2 μm) from days 1 to 2 after infection with a p53 adenovirus. SA‐β‐gal staining was examined at the indicated time points. (H–I) EJ cells expressing an Akt (shAkt) or control (shNC) shRNA were infected with a p53 adenovirus. EJ cells were harvested at the indicated time points and subjected to Western blot analysis using the indicated antibodies (H). SA‐β‐gal staining was performed after 6 days (I). *P <0.05; **P < 0.01 by Student's *t*‐test.

The adenovirus‐mediated expression of p53 has previously been shown to efficiently induce cellular senescence and to be a good model for studying p53‐dependent senescence (Jung *et al*., 2004; Jee *et al*., 2010). Accordingly, the adenovirus‐mediated expression of p53 in EJ p53‐null human bladder cancer cells promoted remarkable morphological changes and senescence‐associated β‐galactosidase (SA‐β‐gal) activity, which are features of premature senescence phenotypes, within 6 days (Fig. 1A,C). Irreversible growth arrest, which is characteristic of cellular senescence, was also confirmed by a marked reduction in cell growth and decreased number of cells in the S‐phase upon p53 expression (Fig. S1A,B). Infection with the control adenovirus (ΔE1) did not alter the cell number, S‐phase, and SA‐β‐gal activity (Fig. S2), confirming that the adenovirus itself did not induce the senescence phenotype in our experimental setting.

Extensive molecular analysis revealed that the levels of Akt phospho‐Ser473 (pS473) and phospho‐Thr308 (pT308), which are both indicators of Akt activation, were strongly increased 2 days after the induction of p53 expression, whereas the Akt protein level remained the same (Fig. 1B). Statistical analysis of the experimental results consistently revealed that Akt activation peaked at 2 days after p53 expression and that it was maintained until the four‐day time point (Fig. 1C). In contrast, the number of SA‐β‐gal‐positive cells was sharply increased at 4 days after p53 expression. These results suggest that Akt is activated during p53‐induced premature senescence prior to the onset of the senescence.

### Akt is required for p53‐induced senescence

The observation that Akt activation occurs prior to the induction of the senescence phenotype raised the possibility that early Akt activation may play a role in p53‐induced senescence. To test this possibility, we treated cells with LY294002, which is the most widely used PI3K/Akt inhibitor, for 2 days starting at three different time points following the induction of p53 expression (Fig. 1D). As shown in Figure 1(E), the Western blotting results revealed that LY294002 treatment from days 0 to 2 (LY 0–2 d) efficiently inhibited Akt activation on day 2, although Akt activation was restored following the removal of LY294002. Similarly, LY294002 treatment from days 2 to 4 (LY 2–4 d) and from days 4 to 6 (LY 4–6 d) also efficiently inhibited Akt activity on days 4 and 6, respectively, whereas Akt activity was not altered at the other time points. Thus, these experimental conditions allowed to us examine the role of Akt activation at different time points. Importantly, treatment with LY294002 from days 0 to 2 after p53 expression inhibited SA‐β‐gal activity more effectively than treatment at later time points (Fig. 1F), suggesting that Akt activation during the early phase is particularly important for inducing the senescence phenotype.

In addition, treatment with the Akt inhibitor IV, another Akt inhibitor that does not interfere with PI3K activity (Kau *et al*., 2003), also significantly suppressed the p53‐induced increase in SA‐β‐gal activity (Fig. 1G). We also examined the role of Akt following the knockdown of Akt expression using an Akt shRNA retrovirus. The Western blotting results demonstrated that the knockdown of Akt expression did not result in the complete suppression of Akt activation (Fig. 1H). Nonetheless, Akt knockdown led to a significant reduction in p53‐induced SA‐β‐gal activity (Fig. 1I). Moreover, p53‐mediated Akt activation and the inhibition of p53‐induced SA‐β‐gal activity by LY294002 treatment were also observed in H1299 human lung cancer cells (Fig. S3). The inhibition of p53‐induced SA‐β‐gal activity by LY294002 or Akt inhibitor IV treatment for up to 12 days did not result in a significant increase in SA‐β‐gal activity (Fig. S4), suggesting that the inhibition of Akt activation did not delay, but rather abrogated, senescence induction. All of these results indicate that Akt activation is required for the p53‐mediated induction of senescence phenotypes.

### Akt and p21 separately regulate p53‐induced ROS increases and cell cycle arrest, respectively

**Figure 2:**
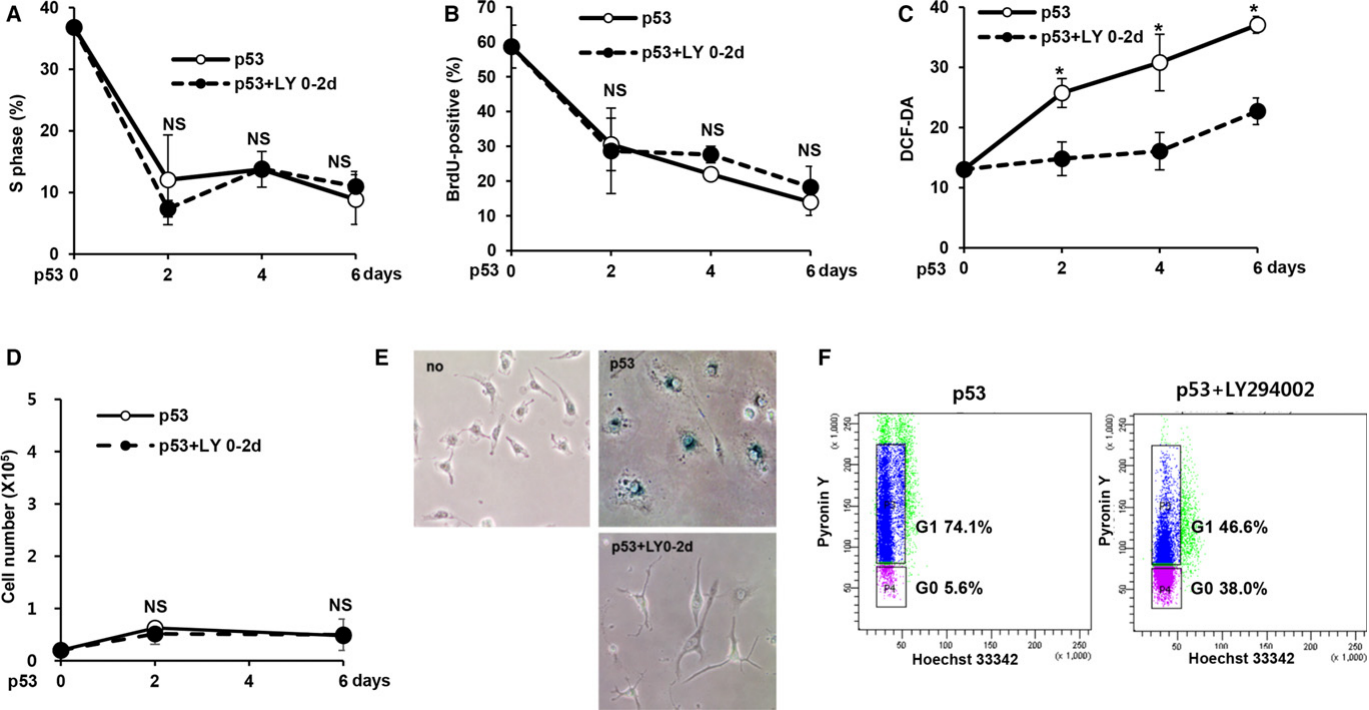
Inhibition of Akt abolished ROS induction but not cell cycle arrest upon p53 expression. (A–F) EJ cells were treated with LY294002 (20 μm) from days 0 to 2 (LY 0–2 d) after infection with a p53 adenovirus. The percentage of cells in the S‐phase was examined using an EPICS XL cytometer (A). BrdU incorporation was assessed using a BrdU staining kit (Invitrogen, Carlsbad, CA, USA) (B). Intracellular ROS levels were measured with an EPICS XL cytometer after the staining of cells with 2′,7′‐dichlorodihydrofluorescein diacetate (DCF‐DA) (50 μm) (C). Cell numbers were determined at the indicated time points by trypan blue exclusion (D). SA‐β‐gal staining was examined after 6 days, and a representative image is shown (E). The percentage of cells in the G0 phase was examined by Pyronin Y/Hoechst 33342 staining (F). *P < 0.05 by Student's *t*‐test. NS, not significant.

We and other groups have previously shown that both cell cycle arrest and increased intracellular ROS levels play important roles in the induction of cellular senescence upon p53 activation (Jung *et al*., 2004; Lu & Finkel, 2008; Jee *et al*., 2010). To explore the role of activated Akt during p53‐induced senescence, we next examined both cell cycle arrest and intracellular ROS levels in LY294002‐treated cells following the induction of p53 expression. Interestingly, LY294002 treatment did not affect the p53‐induced decrease in S‐phase cells or BrdU incorporation (Fig. 2A,B). However, LY294002 cotreatment significantly inhibited the p53‐induced increase in intracellular ROS levels at least until day 4 (Fig. 2C), suggesting that Akt activation is required for the increase in ROS levels during the early phase but not for the induction of cell cycle arrest in response to p53 expression.

We also examined cell proliferation to explore the process of cell cycle arrest in LY294002‐treated cells. LY294002 treatment did not affect the p53‐induced reduction in cell proliferation (Fig. 2D). In addition, cells cotreated with LY294002 did not show any senescence‐associated morphological changes (Fig. 2E). p53 activation has been shown to induce cellular senescence or quiescence, depending on the cell type and physiological conditions (Liebermann *et al*., 2007; Levine & Oren, 2009). The characteristics of p53‐induced quiescence have been reported to include the abrogation of SA‐β‐gal activity, loss of proliferation and nonsenescence morphological features (Korotchkina *et al*., 2010; Leontieva & Blagosklonny, 2010). These results suggest that p53 induces quiescence rather than senescence following Akt inhibition. Consistent with this notion, cells treated with the Akt inhibitor IV and those with Akt knockdown also exhibited a loss of proliferation upon p53 expression (Fig. S5). Furthermore, using Pyronin Y/Hoechst 33342 double staining, a widely used method for distinguishing the G0 from G1 phase (Kim & Sederstrom, 2015), we observed a marked increase in the number of cells in the G0 phase in cells cotreated with LY294002 (Fig. 2F).

**Figure 3:**
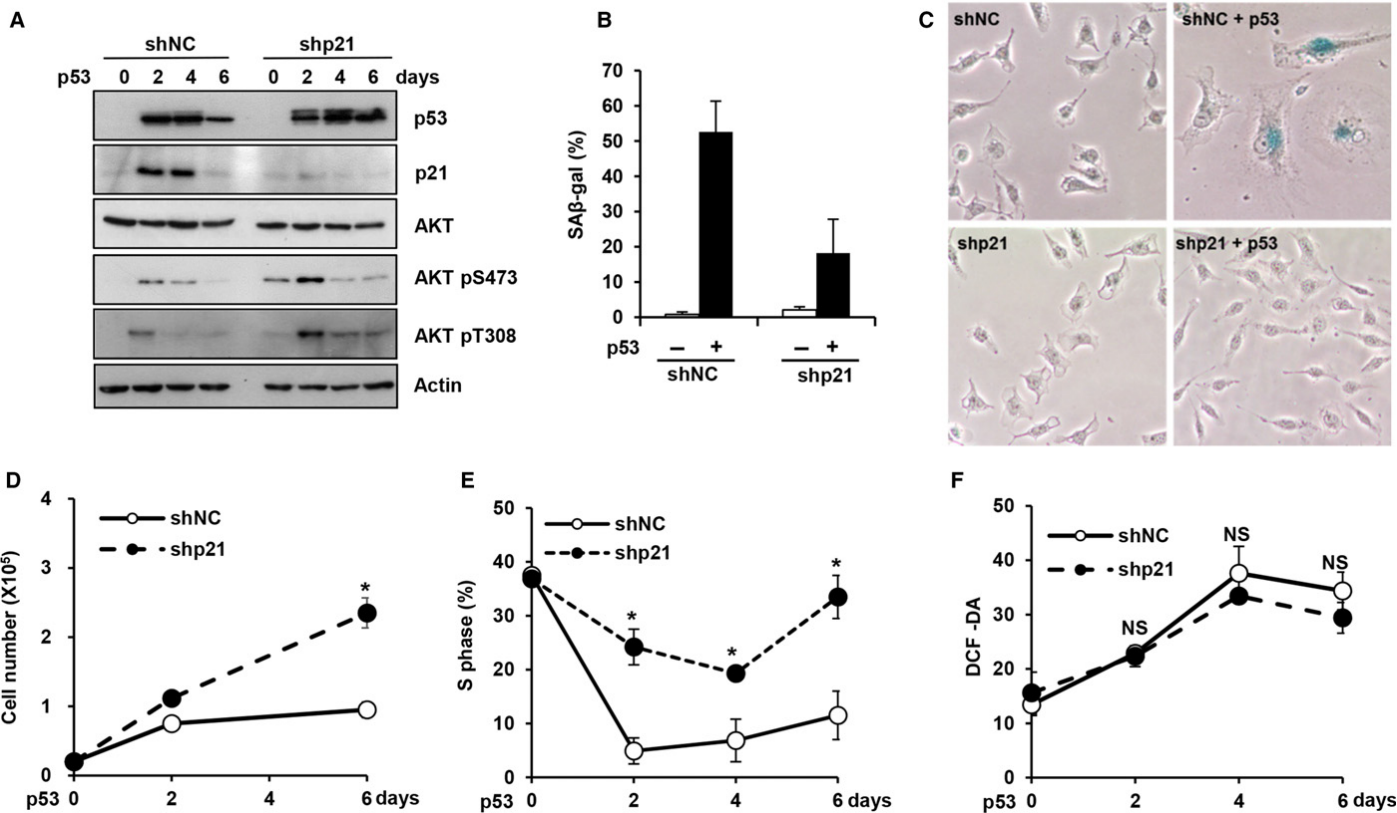
Inhibition of p21 abolished cell cycle arrest but not ROS induction upon p53 expression. (A–F) EJ cells expressing either a p21 shRNA (shp21) or nontargeting shRNA (shNC) were infected with a p53 adenovirus. EJ cells were harvested at the indicated time points and subjected to Western blot analysis using the indicated antibodies (A). SA‐β‐gal staining was performed after 6 days (B), and a representative image is shown (D). Cell numbers were determined at the indicated time points by trypan blue exclusion (D). The percentage of cells in the S‐phase was examined using an EPICS XL cytometer (E). Intracellular ROS levels were measured at the indicated time points using an EPICS XL cytometer after the staining of cells with DCF‐DA (50 μm) (F). *P < 0.05 by Student's *t*‐test. NS, not significant.

The results of our experiments on LY294002‐treated cells raise the possibility that the p53‐induced cell cycle arrest and increase in intracellular ROS levels are separately regulated. To further explore this possibility, we used a p21 shRNA retrovirus to specifically suppress the p53‐mediated induction of p21 expression and confirmed that its expression was effectively suppressed using Western blot analyses (Fig. 3A). As expected, suppression of the induction of p21 expression successfully inhibited the p53‐induced increase in SA‐β‐gal activity and morphological changes (Fig. 3B,C). In addition, the p53‐induced loss of proliferation and cell cycle arrest were inhibited by the suppression of p21 induction (Fig. 3D,E). However, the increase in intracellular ROS levels was not affected by p21 knockdown (Fig. 3F). These results further support the notion that the p53‐induced cell cycle arrest and increase in ROS levels are separately regulated by p21 and Akt, respectively.

### Akt and p21 cooperate in H‐Ras‐induced senescence of normal fibroblasts

**Figure 4:**
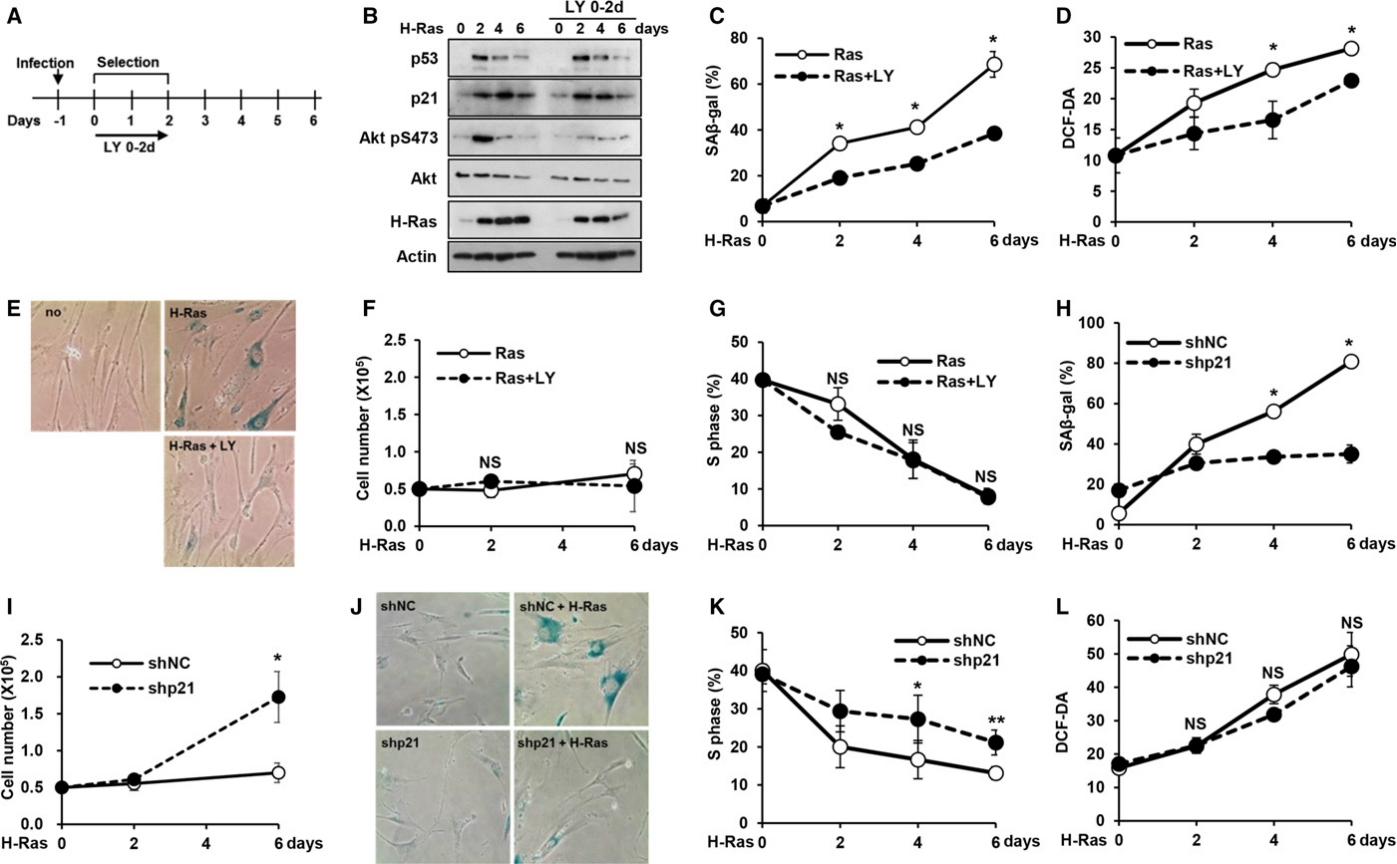
Akt and p21 are independently regulated in H‐Ras‐induced senescence. (A‐G) WI‐38 cells were infected with an H‐RasV12 retrovirus and treated with LY294002 from days 0 to 2 (LY 0–2 d). The experimental procedure for the induction of H‐Ras‐mediated senescence and LY294002 (LY) treatment (A). WI‐38 cells were harvested at the indicated time points and subjected to Western blot analysis using the indicated antibodies (B). SA‐β‐gal staining was examined at the indicated time points (C). Intracellular ROS levels were measured at the indicated time points using an EPICS XL cytometer following the staining of cells with DCF‐DA (50 μm) (D). A representative image of SA‐β‐gal staining captured on the 6th day is shown (E). Cell numbers were determined at the indicated time points by trypan blue exclusion (F). The percentage of cells in the S‐phase was examined using an EPICS XL cytometer (G). (H–L) WI‐38 cells expressing either a p21 (shp21) or nontargeting (shNC) shRNA were infected with an H‐RasV12 retrovirus. SA‐β‐gal staining was performed at the indicated time points (H). Cell numbers were determined at the indicated time points by trypan blue exclusion (I). A representative image of SA‐β‐gal staining captured on the 6th day is shown (J). Intracellular ROS levels were measured at the indicated time points using an EPICS XL cytometer following the staining of cells with DCF‐DA (50 μm) (K). The percentage of cells in the S‐phase was examined using an EPICS XL cytometer (L). *P < 0.05; **P < 0.01 by Student's *t*‐test. NS, not significant.

We next sought to determine whether p21 and Akt are independently regulated in other types of premature senescence. To explore this issue, we examined the roles of Akt and p21 in H‐Ras‐induced senescence in normal WI‐38 fibroblasts. To detect the early activation of Akt, we designated the first day postinfection with the H‐RasV12 retrovirus as day 0, whereas previous studies have set the day after puromycin selection as day 0, which was after H‐Ras retroviral infection (Serrano *et al*., 1997; Mason *et al*., 2004) (Fig. 4A). In this experimental setting, Akt activation and p53 and p21 expression were detected at 2 days after H‐Ras expression, whereas the Akt protein level did not change (Fig. 4B).

In addition, consistent with the results of the experiments on p53‐induced senescence, treatment with LY294002 for 2 days starting on day 0 resulted in the significant suppression of SA‐β‐gal activity, increased ROS levels, and morphological changes upon the induction of H‐Ras expression (Fig. 4C–E). However, neither the H‐Ras‐induced loss of proliferation nor cell cycle arrest was altered (Fig. 4F,G), suggesting that H‐Ras expression under Akt inhibition also induces quiescence.

Conversely, the shRNA‐mediated inhibition of p21 induction resulted in the suppression of SA‐β‐gal activity, loss of proliferation, morphological changes, and cell cycle arrest (Fig. 4H–K). However, p21 knockdown did not suppress the increase in ROS levels upon H‐Ras expression (Fig. 4L). These results further support the notion that the cell cycle arrest and increase in ROS levels are separately regulated. Taken together, our results regarding p53‐ and H‐Ras‐induced senescence show that p53‐dependent senescence is not effectively induced following the inhibition of either Akt or p21.

### Akt induces ROS through the NF‐κB‐NOX4 pathway during p53‐induced senescence

**Figure 5:**
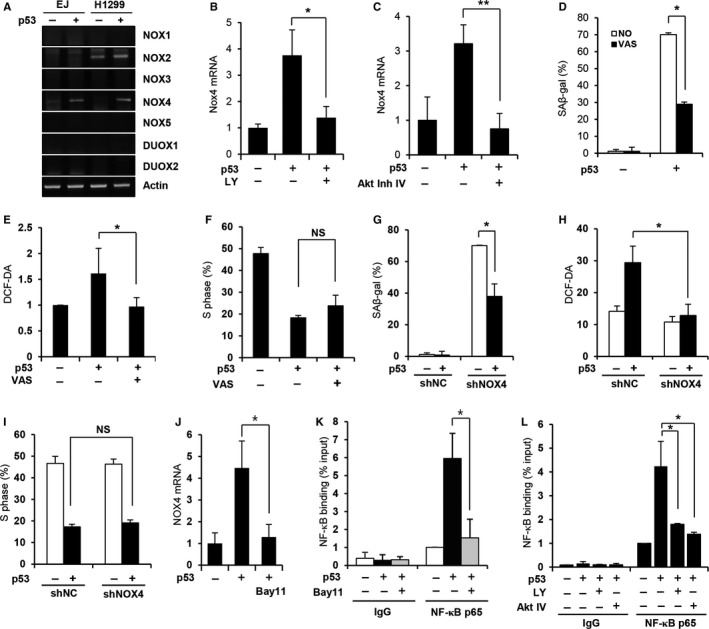
NOX4 mediates the Akt‐dependent increase in ROS levels following the induction of p53 expression. (A) The expression of NADPH oxidases was examined in EJ and H1299 cells using semi‐quantitative RT–PCR with the indicated primers. (B–C) EJ cells were treated with LY294002 (20 μm) for 2 days (LY 0–2 d). (B) or with the Akt inhibitor IV (1.2 μm) from days 1 to 2 (C) after p53 adenovirus infection. Total RNA samples were harvested after 2 days, and the NOX4 mRNA level was determined using real‐time RT–PCR. (D–F) EJ cells were treated with 1 μm VAS2870 (VAS) from days 0 to 2 after infection with a p53 adenovirus. SA‐β‐gal staining was performed after 6 days (D). The intracellular ROS levels (E) and the percentage of cells in the S‐phase (F) were examined. (G–I) EJ cells expressing either a NOX4 (shNOX4) or nontargeting (shNC) shRNA were infected with a p53 adenovirus. SA‐β‐gal staining was performed after 6 days (G). The intracellular ROS levels (H) and the percentage of cells in the S‐phase (I) were determined. (J) EJ cells were treated with Bay11‐7082 (Bay11, 5 μm) from days 1 to 2 after infection with a p53 adenovirus. The NOX4 mRNA level was determined using real‐time RT–PCR. (K–L) ChIP assay for NF‐κB binding to the NOX4 promoter. EJ cells were treated with Bay11‐7082 (5 μm) from days 1 to 2 (K), LY294002 (20 μm) for 2 days, or the Akt inhibitor IV (1.2 μm) from days 1 to 2 (L) after p53 adenovirus infection. The ChIP assay was performed using either the NF‐κB p65 antibody or IgG as a control. *P < 0.05; **P < 0.01 by Student's *t*‐test. NS, not significant.

Considering our finding that Akt plays a critical role in increasing intracellular ROS levels, we next explored the molecular mechanism by which Akt activation controls these levels. ROS levels have been shown to be increased by Akt following a reduction in the MnSOD level through the Akt‐dependent phosphorylation of FOXO (Miyauchi *et al*., 2004; Nogueira *et al*., 2008). However, the MnSOD level was not altered by p53 expression in either EJ or H1299 cells (data not shown). NADPH oxidases (NOXs) are considered important sources of cellular ROS. To gain insights into the roles of NOXs in the Akt‐mediated increase in ROS levels, we examined the levels of NOX family mRNAs in both EJ and H1299 cells following the induction of p53 expression. As shown in Figure 5(A), NOX4 expression was increased by p53 expression. In contrast, NOX2 expression was detected only in H1299 cells and was not significantly altered. Therefore, only NOX4 was further assessed. Real‐time PCR analyses also showed that NOX4 expression was increased by p53 expression in both EJ and H1299 cells. Moreover, the p53‐induced increase in NOX4 expression was abolished by treatment with LY294002 or Akt inhibitor IV (Fig. 5B,C; Fig. S6A), suggesting that Akt activation is required for the p53‐mediated induction of NOX4 expression.

We next examined the role of NOX4 in the p53‐induced increase in ROS levels. Consistent with what was observed following Akt inhibition, treatment with VAS2870, a pan‐NOX inhibitor, efficiently suppressed p53‐induced SA‐β‐gal activity but did not affect the loss of proliferation (Fig. 5D; Fig. S6B). Importantly, VAS2870 treatment suppressed the increase in ROS levels but did not affect cell cycle arrest upon p53 expression in EJ cells (Fig. 5E,F). This VAS2870‐dependent suppression of ROS production was also observed in H1299 cells (Fig. S6C–E), suggesting that NOX4 induction plays a critical role in the Akt‐mediated increase in ROS levels. To further confirm the role of NOX4 in ROS induction, we used an NOX4 shRNA retrovirus to specifically suppress the p53‐mediated induction of NOX4 expression (Fig. S6F). NOX4 knockdown resulted in the significant suppression of p53‐induced SA‐β‐gal activity but did not affect the loss of proliferation (Fig. 5G; Fig. S6G). In addition, NOX4 knockdown abrogated the p53‐induced increase in ROS levels but did not affect cell cycle arrest (Fig. 5H,I). These results further confirm that NOX4 is the downstream regulator of the Akt‐mediated increase in intracellular ROS levels caused by p53 expression.

NOX4 induction by Akt has been recently reported in melanoma (Govindarajan *et al*., 2007) and non‐small cell lung cancer cells (Zhang *et al*., 2014). In addition, Zhang *et al*. reported that Akt induces NOX4 expression through the activation of NF‐κB, a well‐known downstream transcription factor of Akt (Ozes *et al*., 1999). To further explore the molecular mechanism of NOX4 induction in p53‐dependent senescence, we treated cells with BAY 11‐7082 (5 μm), a potent NF‐κB inhibitor, after p53 expression. As shown in Figure 5(J), BAY 11‐7082 completely abolished the p53‐induced increase in NOX4 mRNA. Importantly, the chromatin immunoprecipitation (ChIP) assay results indicated that NF‐κB binding to the putative binding site on the NOX4 promoter (Zhang *et al*., 2014) was enhanced by p53 expression, but this NF‐κB binding was inhibited by BAY 11‐7082 treatment (Fig. 5K). Furthermore, Akt inhibition by either LY294002 or Akt inhibitor IV treatment significantly inhibited the increase in NF‐κB binding in response to p53 expression (Fig. 5L), indicating that Akt activity is required for NF‐κB binding to the NOX4 promoter. These results suggest that Akt induces ROS through the NF‐κB‐NOX4 pathway upon p53 expression.

### Akt activation is mediated by mTOR and is also required for the senescence‐associated secretory phenotype (SASP)

**Figure 6:**
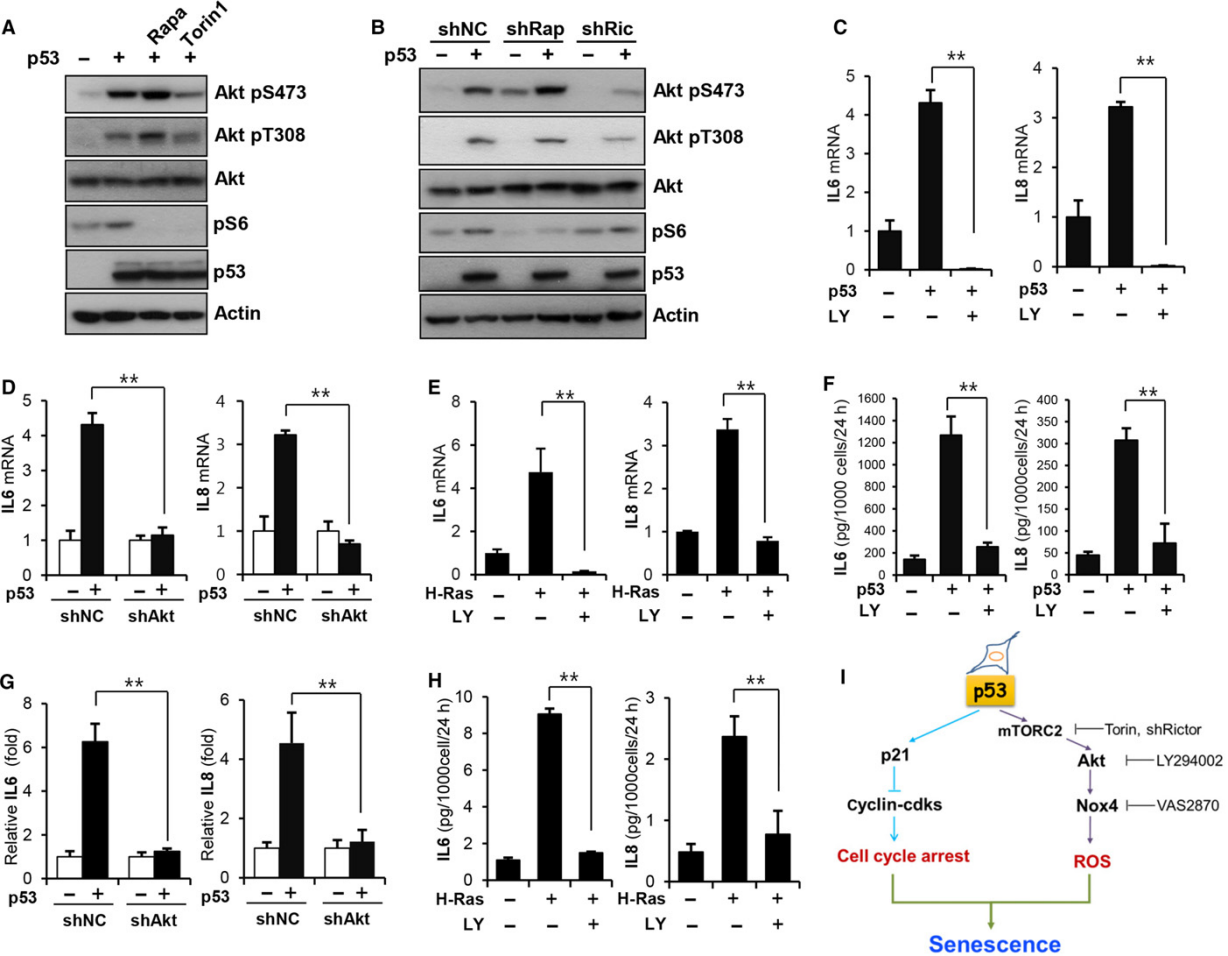
Akt activation is mediated by mTOR and is also required for the SASP. (A) EJ cells were infected with the p53 adenovirus and treated either rapamycin (Rapa, 100 nm) or Torin1 (50 nm) for 2 days before harvested. (B) EJ cells expressing Raptor (shRap), Rictor (shRic), or control (shNC) shRNAs were infected with a p53 adenovirus and harvested 2 days later. Cell lysates were subjected to Western blot analysis using the indicated antibodies. (C–E) Real‐time PCR analysis for IL6 and IL8 mRNA after Akt inhibition. (F–H) ELISA for IL6 and IL‐8 secretion after Akt inhibition. EJ cells were treated with LY294002 (20 μm) for 2 days after p53 adenovirus infection and harvested on day 4 (C, F). EJ cells expressing Akt (shAkt) or control (shNC) shRNAs were infected with the p53 adenovirus and harvested on day 4 (D, G). WI‐38 cells were treated with LY294002 (20 μm) for 2 days after H‐RasV12 retrovirus infection and harvested (E, H). Total RNA samples were subjected to real‐time PCR, and the supernatants were analyzed for the secretory levels of IL6/IL8 by ELISA. (I) A model depicting the cooperative activity between Akt and p21 in inducing cellular senescence. **P < 0.01 by Student's *t*‐test.

We next explored how p53 activates Akt. It has been previously shown that the activation of Akt is achieved by the phosphorylation of Thr308 by PDK‐1 and the phosphorylation of Ser473 by mTORC2. Interestingly, we observed increased phosphorylation of ribosomal S6 protein, a well‐known marker for mTOR activation, upon p53 expression (Fig. 6A). To examine whether mTOR is involved in p53‐mediated Akt activation and the roles of the mTOR‐containing multisubunit complexes, mTORC1 and mTORC2, we treated cells with the mTORC1 inhibitor rapamycin (100 nm) and the mTORC1/mTORC2 inhibitor Torin1 (50 nm) upon p53 expression. Torin1 treatment inhibited p53‐mediated Akt Ser473 phosphorylation (Fig. 6A). Akt Ser473 phosphorylation was slightly increased after rapamycin treatment, most likely due to the loss of mTORC1‐mediated negative feedback on Akt (O'Reilly *et al*., 2006; Zhang *et al*., 2007; Carracedo *et al*., 2008). These results suggest that activation of mTORC2 may be required for Akt activation upon p53 expression. Consistent with this notion, the inactivation of mTORC2 by Rictor knockdown (Fig. S7A) abolished p53‐mediated Akt Ser473 phosphorylation, whereas the inactivation of mTORC1 by Raptor knockdown (Fig. S7B) markedly increased Akt Ser473 phosphorylation (Fig. 6B). These results further confirm that mTORC2 is responsible for Akt activation upon p53 expression.

The SASP has recently been shown contribute to multiple biological functions such as tumor suppression, tissue repair, and aging, through either the autocrine or paracrine mechanisms of various secretory factors including IL6 and IL8 (Tchkonia *et al*., 2013). To examine whether Akt is also involved in the SASP, we examined the effect of Akt inhibition on IL6 and IL8 levels, representative SASP genes, upon p53 expression using quantitative RT–PCR. Interestingly, either LY294002 treatment or Akt knockdown completely abolished the p53‐induced induction of IL6 and IL8 mRNA (Fig. 6C,D). LY294002 treatment also abolished the H‐Ras‐induced induction of IL6 and IL8 mRNA (Fig. 6E). These results suggest that Akt inhibition prevents the induction of the SASP. The requirement of Akt for the SASP was further confirmed by measuring the secreted IL6 and IL8 levels using enzyme‐linked immunosorbent assay (IL6 and IL6 ELISA kits, Peprotech, Rocky Hill, NJ, USA). Consistent with the quantitative RT–PCR results, Akt inhibition also abolished p53‐ and H‐Ras‐mediated IL6 and IL8 secretion (Fig. 6F–H). These results suggest that Akt is also required for the induction of the SASP, at least in p53‐dependent cellular senescence.

## Discussion

In this study, we have provided evidence that cell cycle arrest and increased ROS levels are regulated separately by p21 and Akt, respectively, during p53‐dependent premature senescence. Although p21 is known to be essential for p53‐induced cell cycle arrest, this is the first report that Akt is required for the p53‐induced increase in intracellular ROS levels. These separate roles in cell cycle arrest and ROS induction were also demonstrated during H‐Ras‐induced senescence in normal human WI‐38 fibroblasts. These data suggest that Akt and p21 cooperation is involved in a common mechanism underlying the induction of p53‐dependent premature senescence (Fig. 6I). The necessity of cooperation among more than two regulators for the induction of cellular senescence has been previously reported in a variety of cellular contexts. For example, the coexpression of Ras or Mek1 together with p53 is required to induce senescence in p53‐null MEFs (Ferbeyre *et al*., 2002). The Raf and Akt pathways have been shown to cooperatively control the onset of senescence in response to anticancer drug and hormonal treatments (Taylor *et al*., 2011). Therefore, our and other researchers’ results suggest that cellular senescence is induced by the contributions of multiple signaling pathways and that this induction is dependent on the cellular context rather than on a specific linear pathway.

Akt has been previously implicated in various types of cellular senescence. Miyauchi *et al*. have reported that Akt activation promotes a senescence‐like phenotype in endothelial cells via a p53‐dependent pathway (Miyauchi *et al*., 2004). Additionally, Nogueira *et al*. have demonstrated that Akt‐deficient MEFs are resistant to both replicative and premature senescence (Nogueira *et al*., 2008). Moreover, these studies have also revealed that the increase in intracellular ROS levels following Akt activation is important for the induction of cellular senescence (Miyauchi *et al*., 2004; Nogueira *et al*., 2008). Consistent with this notion, we found that the inhibition of Akt activation using LY294002 or the Akt inhibitor IV or the knockdown of Akt expression suppressed the p53‐induced increase in ROS levels and SA‐β‐gal activity in both EJ and H1299 cancer cells. Furthermore, the inhibition of Akt activation also suppressed the increase in ROS levels and SA‐β‐gal activity during H‐Ras‐induced senescence in WI‐38 cells. These results suggest that Akt contributes to the induction of senescence by promoting an increase in intracellular ROS levels.

We noticed Akt inhibition did not abrogate the p53‐induced loss of cell proliferation, whereas the suppression of p21 induction resulted in the recovery of cell proliferation. Previous studies have shown that p53 activation results in apoptosis, reversible cell cycle arrest (quiescence) or irreversible growth arrest (senescence), depending on the cell type and physiological conditions (Liebermann *et al*., 2007; Levine & Oren, 2009). Recently, a series of studies conducted by Blagosklonny's group have shown that p53 activation induces reversible or irreversible growth arrest, depending on the cellular context (Korotchkina *et al*., 2010; Leontieva & Blagosklonny, 2010; Leontieva *et al*., 2011). These studies suggested that ROS levels and mTOR activity are important following p53 activation (Vigneron & Vousden, 2010). The findings of these studies also suggest that p53 induces reversible cell cycle arrest (quiescence) in the presence of low levels of ROS and mTOR activity. Interestingly, we observed that p53 expression resulted in the loss of proliferation, nonsenescence morphological features, and the abrogation of SA‐β‐gal activity, which are characteristics of quiescence, in the presence of the Akt inhibitor LY294002. Similar results were observed following cotreatment with the Akt inhibitor IV and Akt knockdown. These results indicated that p53 expression induced quiescence following Akt inhibition. Consistent with this notion, we also observed that H‐Ras expression resulted in similar changes in cells cotreated with LY294002. The findings of the present study have shown that the inhibition of Akt or p21 results in different effects, suggesting that ROS and cell cycle arrest may play different roles in the induction of cellular senescence.

The results of the current study have revealed that Akt regulates the intracellular ROS levels through NOX4. A functional link between the PI3K/Akt pathway and NOX family enzymes in the context of intracellular ROS regulation has been suggested in previous studies (Govindarajan *et al*., 2007; Zhang *et al*., 2014). Consistent with the results of these studies, our results showed that the NOX4 mRNA level was increased following the induction of p53 expression and that this increase was dependent upon Akt. We also showed that NOX4 was essential for p53‐mediated ROS induction. Interestingly, p53‐induced cell cycle arrest was not abrogated following NOX4 inhibition, confirming that the regulation of cell cycle arrest and induction of ROS during p53‐induced senescence are independent processes. Collectively, the results of the present study have established a functional link between NOX4‐ and p53‐induced cellular senescence for the first time.

Regarding the molecular mechanism by which Akt induces NOX4 expression, Zhang *et al*. have previously shown that inhibition of the transcription factor NF‐κB abrogates the effect of Akt on NOX4 expression (Zhang *et al*., 2014). Moreover, they have demonstrated that NF‐κB directly binds to the NOX4 promoter and that Akt inhibition reduces NF‐κB binding to the NOX4 promoter. Consistent with these results, treatment with the NF‐κB inhibitor Bay 11‐7082 abolished p53‐induced NOX4 induction. Moreover, we found that p53 induced the direct binding of NF‐κB to the NOX4 promoter, and the inhibition of Akt by LY294002 or Akt inhibitor IV treatment abolished NF‐κB binding to the NOX4 promoter upon p53 expression. Akt has been reported to activate the transcriptional function of NF‐κB through the phosphorylation of IKK (IκB) kinase (Ozes *et al*., 1999). Thus, our results suggest that Akt induces NOX4 expression through the activation and direct binding of NF‐κB to the NOX4 promoter upon p53 expression. Interestingly, p38 has also been reported to play important roles in increasing ROS levels and inducing senescence in response to p53 expression (Jung *et al*., 2004; Kang *et al*., 2005). Thus, it is possible that Akt triggers an increase in ROS levels by inducing NOX4 during the early phase of cellular senescence, whereas p38 plays a role in maintaining ROS levels during a relatively later phase. Interestingly, we observed an increase in ROS levels on day 6 of p53 expression following cotreatment with LY294002 from days 0 to 2. Whereas the increase in ROS levels during the early phase was suppressed by the inhibition of Akt activation, ROS could be induced during the later phase by another factor, such as p38. The functional and kinetic relationships between Akt and p38 remain to be further explored.

Macip *et al*. previously reported that the overexpression of p21 induced the production of intracellular ROS and cellular senescence in EJ cells (Macip *et al*., 2002). Many studies have explored the effect of p21 on ROS production, but the results have varied depending on the cell type, suggesting that the expression level of p21 and cellular context are possibly important. Consistent with this notion, Inoue *et al*. showed that the expression of p21 resulted in either cell death or senescence depending on the levels of p21‐induced ROS (Inoue *et al*., 2009). Fitzgerald *et al*. showed that persistent ROS elevation, accompanied by the long‐term expression of p21, is required to induce senescence of head and neck squamous cell carcinoma (HNSCC) cells (Fitzgerald *et al*., 2015). In addition, the cellular context including the status of p53 also influences the effect on p21 on ROS regulation. Importantly, the suppression of p53 expression inhibited p21‐induced cellular responses (Inoue *et al*., 2009; Fitzgerald *et al*., 2015), suggesting that the p53 status is an important factor for determining the effect of p21. Thus, the discrepancy regarding the role p21 in ROS regulation between Macip's study and our study is possibly due to the different experimental settings or due to pleiotropic role of p21 in different cellular contexts. It is possible that p21 only regulates cell cycle arrest in the presence of p53, whereas in the absence of p53, p21 regulates both cell cycle arrest and ROS production. The precise role of p21 in ROS regulation during p53‐induced senescence should be explored in additional studies.

Regarding how p53 activates Akt during cellular senescence, we observed that mTOR is activated in response to p53 expression and mTORC2 is required for p53‐dependent Akt activation. Although phosphorylation at Thr308 by PDK‐1 induced a structural change of Akt, subsequent mTORC2‐dependent phosphorylation of Ser473 is important for the stabilization and full activation of Akt (Sarbassov *et al*., 2005). In this study, whereas treatment with the mTORC1 inhibitor rapamycin slightly increased p53‐mediated Akt Ser473 phosphorylation, the mTORC1/mTORC2 inhibitor Torin1 suppressed Ser473 phosphorylation. The essential role of mTORC2 in p53‐induced Akt activation was further confirmed by the knockdown of Rictor, a binding partner of mTOR in the mTORC2 complex, indicating that p53 activates Akt through mTORC2. mTOR has been implicated in aging and senescence. Aberrant elevation of mTOR activity has been reported in aging tissue, and the inhibition of mTOR has been shown to increase lifespan. Although mTORC1 is thought to regulate autonomous cell growth, mTORC2 is thought to control proliferation and survival. We previously showed that mTORC2 also plays an important role in cancer cell metastasis (Kim *et al*., 2011). In the present study, we showed that mTORC2 is involved in p53‐induced senescence. The precise role of mTORC2 in cellular senescence and aging needs to be explored in further studies.

We also found that Akt activation is important for the SASP. The inhibition of Akt activation using LY294002 or Akt knockdown suppressed both the mRNA induction and secretion of IL6 and IL8 in both p53‐induced senescent EJ cells and H‐Ras‐induced senescent WI‐38 cells. The SASP has been recently shown to play a critical role in the function of cell senescence and aging itself; thus, identifying the regulatory mechanism of the SASP is emerging as an important research topic. We showed here, for the first time, that Akt plays a critical role in the development of the SASP, at least during p53‐induced senescence. Further studies investigating the precise role of Akt on the induction of the SASP will provide valuable information about the signaling pathway responsible for the SASP and novel targets to control the SASP. Interestingly, NF‐κB is a master transcription regulator for many SASP factors including IL6 and IL8 (Malaquin *et al*., 2016). Therefore, it is possible that Akt regulates both ROS generation and the SASP simultaneously through NF‐κB. Whether Akt‐dependent activation of NF‐κB is also required for the SASP should be determined.

In conclusion, considering the results of the present study, we propose that Akt and p21 play cooperate roles in the p53‐mediated induction of cellular senescence and that complex interactions between signaling pathways occur during this process.

## Experimental procedures

Detailed descriptions of the experimental procedures, reagents, and associated references can be found in Appendix S1 (Supporting information).

## Funding

This work was supported by a grant from National Research Foundation of Korea (2016R1A2B2008887) and Medical Research Center grant (2016R1A5A2007009).

## Author contributions

H.J.J., Y.Y.K., and J.Y. initiated and designed the study. Y.Y.K., H.J.J., and J.U. performed the experiments and analyzed and interpreted the data. J.U., Y.M.K., and S.S.B. conceived the specific experiments and participated in writing the manuscript draft. H.J.J., Y.Y.K., and J.Y. wrote the manuscript. All authors reviewed the manuscript and provided editorial input.

## Conflict of interest

None declared.

## Supporting information


**Fig. S1** Effect of p53 expression on cell proliferation and S phase cells.
**Fig. S2** Effect of infection with the control adenovirus (ΔE1).
**Fig. S3** Akt activation is required for p53‐induced senescence in H1299 cells.
**Fig. S4** Effect of Akt inhibition on p53‐induced cellular senescence.
**Fig. S5** Effect of Akt inhibition on cell proliferation upon p53 expression.
**Fig. S6** NOX4 is responsible for the Akt‐induced increase in ROS levels.
**Fig. S7** Confirmation of Raptor and Rictor knockdown.
**Table S1** Gene‐specific primer sequences used for RT‐PCR.
**Appendix S1** Experimental procedures.
