# Supplementary material for: Cooperation between p21 and Akt is required for p53‐dependent cellular senescence
Source: Aging Cell. 2017 Jul 9;16(5):1094–103. doi: 10.1111/acel.12639 (PMC5595696; doi:10.1111/acel.12639)
Supplement: Supplementary file 1 — Fig. S1 Effect of p53 expression on cell proliferation and S phase cells. Fig. S2 Effect of infection with the control adenovirus (ΔE1). Fig. S3 Akt activation is required for p53‐induced senescence in H1299 cells. Fig. S4 Effect of Akt inhibition on p53‐induced cellular senescence. Fig. S5 Effect of Akt inhibition on cell proliferation upon p53 expression. Fig. S6 NOX4 is responsible for the Akt‐induced increase in ROS levels. Fig. S7 Confirmation of Raptor and Rictor knockdown. Table S1 Gene‐specific primer sequences used for RT‐PCR. Appendix S1 Experimental procedures. [file ACEL-16-1094-s001.docx]

**SUPPLEMENTARY INFORMATION**

**Cooperation between p21 and Akt is required for p53-dependent cellular senescence.**

Young Yeon Kim^1,2,*^, Hye Jin Jee^1,2,*^, Jee-Hyun Um^1,2*^, Young Mi Kim^1,2^, Sun Sik Bae^3^ and Jeanho Yun^1,2^

1 Peripheral Neuropathy Research Center, and 2 Department of Biochemistry, College of Medicine, Dong-A University, Busan, 49201, 3Department of Pharmacology, School of Medicine, Pusan National University, Yangsan-si, 602-739, Republic of Korea.

*These authors contributed equally to this work.

# **Experimental procedures**

**Cell lines, senescence induction and treatments**

EJ human bladder carcinoma cells, H1299 human lung cancer cells, and WI-38 human normal fibroblast cells were maintained in DMEM containing 10% fetal bovine serum (FBS; JR Scientific Inc., Woodland, CA, USA). To induce p53-induced premature senescence, EJ and H1299 cells were infected with a recombinant adenovirus encoding the wild-type p53 or control adenovirus (ΔE1) at 100 MOI for 3 h, as previously described ([Jee *et al.*, 2010](#_ENREF_1)). To promote H-Ras-induced senescence, a retroviral H-rasV12 construct (Addgene plasmid #1768) was transfected into ecotropic BOSC cells, and pantropic retroviral packaging constructs and cell-free viral supernatants were used to infect WI-38 cells. The first day after H-Ras retroviral infection was designated as day 0 in all experiments. Following retroviral infection, cells were selected with puromycin (2 μg/ml) for two days. LY294002 and Torin1 were purchased from Calbiochem (EMD Millipore, Billerica, MA, USA). The Akt inhibitor IV and VAS2870 were obtained from Santa Cruz Biotechnology (Santa Cruz, CA, USA). Bay 11-7082 and rapamycin were purchased from Sigma-Aldrich (St. Louis, MO, USA).

**SA-β-gal staining**

Cells were fixed in 0.25% glutaraldehyde, and SA-β-gal staining was performed at pH 6.0. Following cell staining, at least 300 cells were examined in several fields, and SA-β-gal-positive cells were counted. These experiments were repeated three times, and the results are presented as the mean and standard deviation.

**Cell proliferation assay**

To assess cell proliferation, 1×10^4^ cells were seeded into 6-well plates. The numbers of cells were counted using a LUNA automated cell counter (Logos Biosystems Inc., Anyang, South Korea), according to the manufacturer’s recommendation, at the indicated time points after staining with trypan blue. For each experiment, each time point was assessed in triplicate, and the results are presented as the mean and standard deviation.

**Western blot analysis and antibodies**

Cells were lysed in RIPA buffer and subjected to Western blot analysis. The anti-p53 and anti-NF-κB p65 antibodies were obtained from Santa Cruz Biotechnology (Santa Cruz, CA, USA). In addition, an anti-p21 antibody was purchased from Oncogene Science (Uniondale, NY, USA). The anti-Akt, anti-Akt pS473, anti-Akt pT308, and anti-phospho-S6 (Ser235/256) antibodies were obtained from Cell Signaling Technology (Danvers, MA, USA), and an anti-H-Ras antibody was purchased from BD Biosciences (San Jose, CA, USA). The actin level was monitored as an internal loading control using an anti-actin (Sigma-Aldrich) antibody. All Western blot analyses were repeated three times. Band intensities were quantified using densitometry and AlphaEaseFC 4.0 software (Alpha Innotech Corp., San Leandro, CA, USA). Relative band intensities were calculated by normalizing the band intensities for the untreated samples to that of the actin signal.

**RNA interference**

To create EJ cell lines in which Akt expression was knocked down, a lentiviral construct ([Kim *et al.*, 2011](#_ENREF_3)) encoding an shRNA against Akt1 was inserted into a pLKO.1 plasmid and transfected into 293FT packaging cells. The resulting cell-free viral supernatant was used to infect EJ cells. Retroviral constructs containing a p21 or NOX4 shRNA were gifted by Dr. Pan Zhang (University of Michigan, Ann Arbor, MI) and Dr. Lance Terada (University of Texas Southwestern Medical Center, Dallas, TX), respectively. The retroviral constructs were transfected into BOSC cells together with pantropic retroviral packaging constructs, and the resulting cell-free viral supernatants were used to infect EJ and H1299 cells. To knockdown Raptor and Rictor, lentivirus constructs containing Raptor and Rictor shRNA (kindly provided by Dr. Mee-Sup Yoon, Gachon University School of Medicine, Incheon, Korea) were transfected into 293FT packaging cells and the resulting cell-free viral supernatant was used to infect EJ cells. After puromycin selection, resistant cells were pooled and used for the remaining experiments.

**Cell cycle analysis and BrdU incorporation assay**

Cell cycle analysis was performed after propidium iodide (PI) staining using an EPICS XL cytometer and WINCYCLE software (Beckman Coulter, Inc., Indianapolis, IN, USA), as described previously ([Jee *et al.*, 2013](#_ENREF_2)). A total of 10,000 events were analyzed for each sample, and the experiment was repeated at least three times.

To analyze the G0 and G1 phase cells, Pyronin Y/Hoechst 33342 double staining was performed as previously described ([Kim & Sederstrom, 2015](#_ENREF_4)). Briefly, cells were harvested and fixed with 70% ethanol. After washing, the cells were stained with the staining solution containing 4 μg/ml of Pyronin Y (Sigma) and 2 μg/ml of Hoechst 33342 in PBS for 20 min at room temperature. The stained cells were analyzed with a FACSCalibur flow cytometer (BD Bioscience) with a 355 nm and 488 nm laser.

BrdU incorporation assays were performed using a BrdU Staining Kit (Invitrogen, Waltham, MA, USA) according to the manufacturer’s instructions. Following cell staining, at least 300 cells were examined in each of several fields, and BrdU-positive cells were counted. These experiments were repeated three times, and the results are presented as the mean and standard deviation.

**Measurement of intracellular ROS levels**

To measure intracellular ROS levels, cells were stained with 50 μM dichlorodihydrofluorescein diacetate (DCF-DA) (Sigma-Aldrich Co., Ltd) for 30 min and then harvested. Fluorescence intensities were quantified using an EPICS XL cytometer (Beckman Coulter, Inc.). Experiments were performed in triplicate, and the results are presented as the mean and standard deviation.

**Quantitative RT-PCR**

For semi-quantitative reverse transcription (RT)-PCR analyses, cDNA was synthesized from total RNA as described previously ([Jee *et al.*, 2010](#_ENREF_1)). PCR was performed in HiPi PCR Premix (ELPIS, Daejeon, South Korea) using cDNA as the template. PCR products were separated by electrophoresis in 2% agarose gels. The following PCR conditions were used: one cycle at 95°C for 5 min, followed by 22 cycles at 95°C for 45 sec, 55°C for 1 min, and 72°C for 1 min. All experiments were repeated at least three times.

Quantitative real-time PCR was performed in triplicate in SYBR Green PCR Master Mix (Takara Bio Inc., Shiga, Japan) with an ABI Prism 7500 Real-time PCR System (Applied Biosystems). β-actin was used as an internal control for all samples, with normalization of gene-specific mRNA levels to the β-actin RNA level. The level of each mRNA was determined using the 2^-ΔCΤ^-threshold cycle method. The PCR primers used are listed in Table S1 (Supporting Information).

**Chromatin immunoprecipitation (ChIP) assay**

ChIP assay was performed using Pierce Agarose ChIP Kit (Thermo scientific, Waltham, MA, USA), according to the manufacturer’s instructions. Briefly, 1 X 10^7^ cells were cross-linked with 1% formaldehyde for 10 min. After the cells were lyased, DNA was digested with micrococcal nuclease and the digested chromatin was immunoprecipitated with 5 μg of the NF-κB p65 antibody (Santa Cruz) or normal mouse IgG (Millipore 12-371). The immunoprecipitates were washed with low- and high-salt wash buffers, de-cross-linked and eluted. DNA was purified with DNA cleanup columns and analyzed by quantitative real-time PCR (as described above) using primers for NF-κB binding element on NOX4 promoter ([Zhang *et al.*, 2014](#_ENREF_5)): forward 5’- GCTTTAGTTTGGGAGTGGGA -3’ reverse 5’- GAAATTTGAGCCGGAAACAG -3’.

**IL6 and IL8 ELISA**

Conditioned medium was generated by adding fresh DMEM without FBS to cells for 24 h and then the cells were trypsinized and counted. The secreted level of IL6 and IL8 was determined using IL6 and IL8 ELISA kits (Peprotech, Rocky Hill, NJ, USA), according to the manufacturer’s protocol. The concentrations of IL6 and IL8 were calculated according to the standard curves, which were generated by the standard mixtures provided with each assay kit. All samples were run in triplicated and the results were normalized to the total cell number.

**Statistical analysis**

All data are presented as the mean ± standard deviation. Differences between the various experimental groups were examined using Student’s *t*-test. A *P*-value of < 0.05 was considered statistically significant.

**References for Experimental Procedures**

Jee HJ, Kim AJ, Song N, Kim HJ, Kim M, Koh H*, et al.* (2010) Nek6 overexpression antagonizes p53-induced senescence in human cancer cells. *Cell Cycle* **9**, 4703-4710.

Jee HJ, Kim HJ, Kim AJ, Song N, Kim M, Lee HJ*, et al.* (2013) The inhibition of Nek6 function sensitizes human cancer cells to premature senescence upon serum reduction or anticancer drug treatment. *Cancer Lett*. **335**, 175-182.

Kim EK, Yun SJ, Ha JM, Kim YW, Jin IH, Yun J*, et al.* (2011) Selective activation of Akt1 by mammalian target of rapamycin complex 2 regulates cancer cell migration, invasion, and metastasis. *Oncogene* **30**, 2954-2963.

Kim KH, Sederstrom JM (2015) Assaying Cell Cycle Status Using Flow Cytometry. *Curr. Protoc. Mol. Biol*. **111**, 28 26 21-11.

Zhang C, Lan T, Hou J, Li J, Fang R, Yang Z*, et al.* (2014) NOX4 promotes non-small cell lung cancer cell proliferation and metastasis through positive feedback regulation of PI3K/Akt signaling. *Oncotarget* **5**, 4392-4405.

**Supplementary Figures**

**
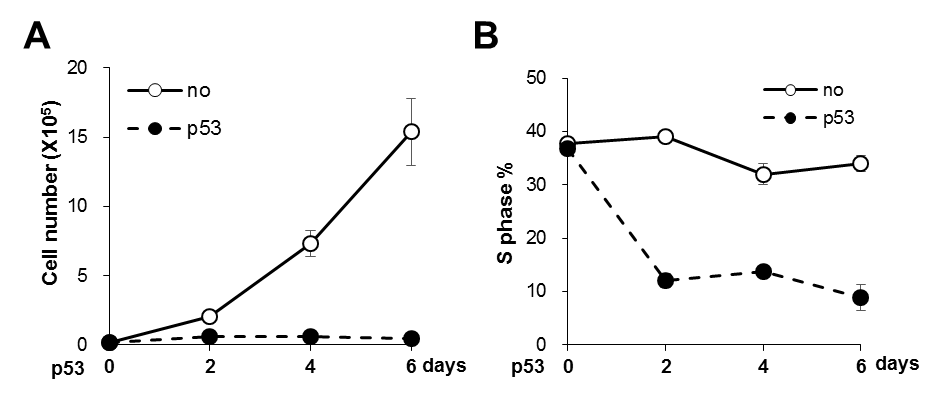
**

**Fig. S1**. Effect of p53 expression on cell proliferation and S phase cells.

EJ cells were infected with a p53 adenovirus at 100 MOI. (A) Cell numbers were determined using a LUNA automated cell counter at the indicated time points by trypan blue exclusion. (B) The percentage of cells in S phase was examined using an EPICS XL cytometer.

**
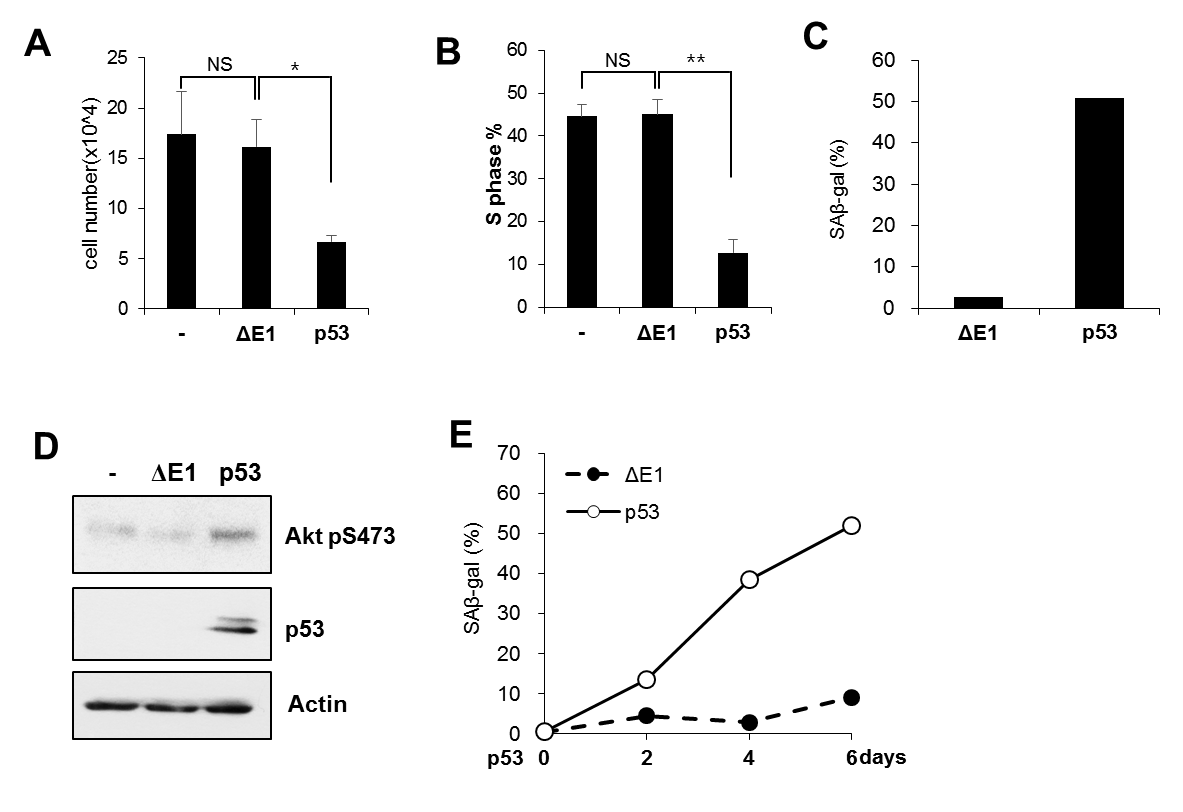
**

**Fig. S2**. Effect of infection with the control adenovirus (ΔE1).

(A-D) EJ cells were infected with a recombinant adenovirus encoding the wild-type p53 or control adenovirus (ΔE1) at 100 MOI for 3 h. The cell number (A) and percentage of S phase cells (B) were examined four days later. (C) SA-β-gal staining was performed after 6 days. (D) Cells were harvested two days later, and the cell lysates were subjected to Western blot analysis using the indicated antibodies. (E) H1299 cells were infected with a recombinant adenovirus encoding the wild-type p53 or control adenovirus (ΔE1) at 100 MOI for 3 h and SA-β-gal staining was performed at the indicated time points. **P* <0.05; ***P* <0.01 by Student’s *t*-test. NS, not significant.

**
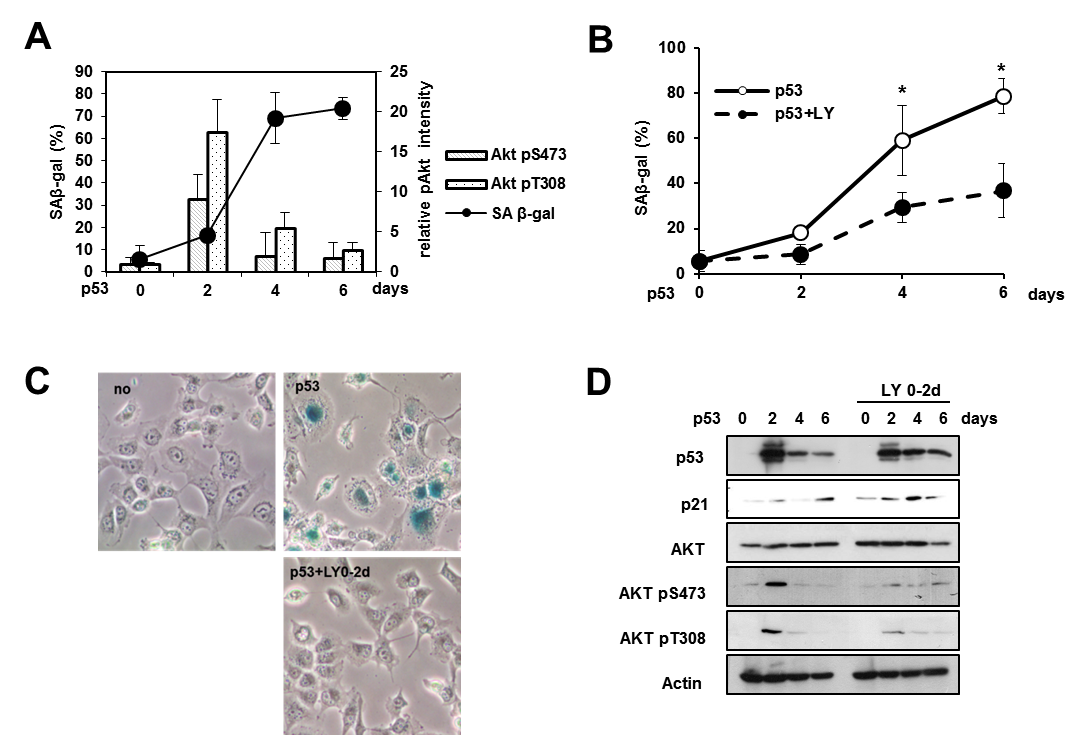
**

**Fig. S3**. Akt activation is required for p53-induced senescence in H1299 cells.

(A) H1299 cells were infected with a p53 adenovirus at 100 MOI. SA-β-gal staining and Western blot analysis were performed using anti-Akt pS473 and pT308 antibodies at the indicated time points. The percentages of SA-β-gal-positive cells were determined at the indicated time points after p53 expression and plotted against the levels of Akt pS473 and pT308. (B-D) H1299 cells were treated with LY294002 (20 μM) from days 0 to 2 (LY 0-2 d) after infection with a p53 adenovirus. SA-β-gal staining was examined at the indicated time points. The percentage of SA-β-gal-positive cells was plotted (B). Representative images of SA-β-gal staining were captured after six days and are shown in (C). The results of Western blot analysis of cell lysates harvested at the indicated time points (D). *P <0.05 by Student’s t-test.


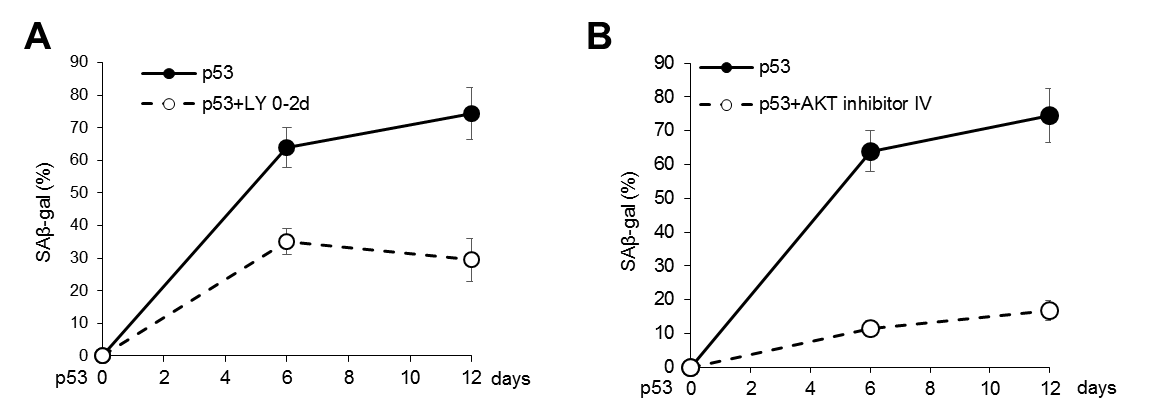


**Fig. S4.** Effect of Akt inhibition on p53-induced cellular senescence.

EJ cells were treated with LY294002 (20 μM) (LY 0-2 d) from days 0 to 2 (A) or the Akt inhibitor IV (1.2 μM) from days 1 to 2 (B) after infection with a p53 adenovirus. SA-β-gal staining was performed after 6 and 12 days.

**
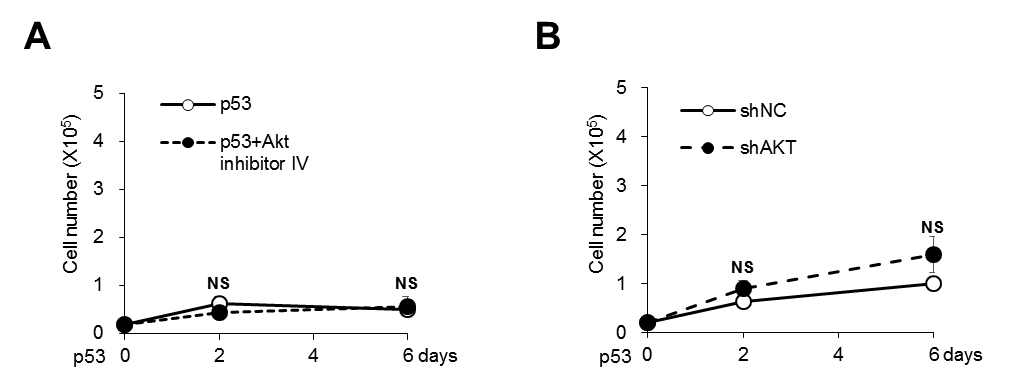
**

**Fig. S5.** Effect of Akt inhibition on cell proliferation upon p53 expression.

EJ cells were treated with the Akt inhibitor IV (1.2 μM) from days 1 to 2 after infection with a p53 adenovirus (A). EJ cells expressing an Akt (shAkt) or control (shNC) shRNA were infected with the p53 adenovirus (B). Cell numbers were determined at the indicated time points by trypan blue exclusion using a LUNA automated cell counter. NS, not significant.

**
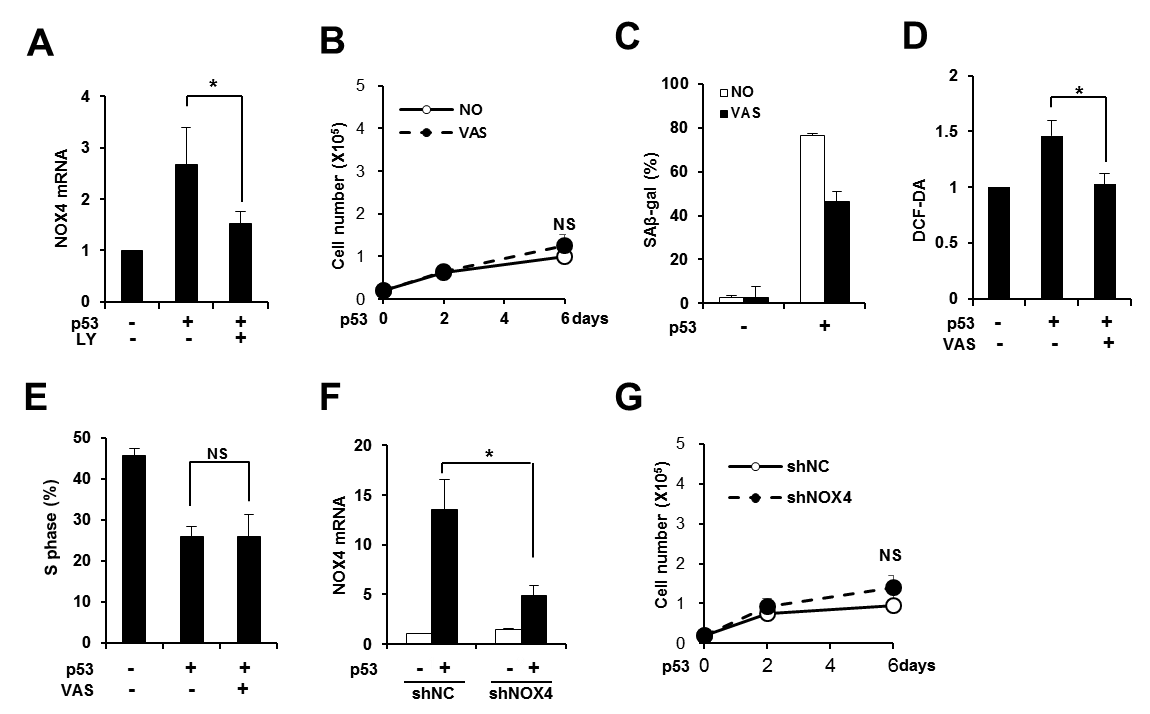
**

**Fig. S6**. NOX4 is responsible for the Akt-induced increase in ROS levels.

(A) H1299 cells were treated with LY294002 (20 μM) for 2 days (LY 0-2 d) after p53 adenovirus infection. Total RNA samples were harvested after two days, and the NOX4 mRNA level was determined using real-time RT-PCR. (B-E) H1299 cells were treated with 1 μM VAS2870 (VAS) for two days after p53 induction. Cell numbers at the indicated time points were examined (B). (C) SA-β-gal staining was performed after 6 days. Intracellular ROS levels (D) and the percentage of cells in S phase (E) were examined after two days. (F) The mRNA levels of NOX4 in EJ cells expressing either an NOX4 shRNA (shNOX4) or non-targeting shRNA (shNC) were determined using real-time RT-PCR. (G) Cell numbers at the indicated time points were examined. *P <0.05 by Student’s t-test NS, not significant.

**
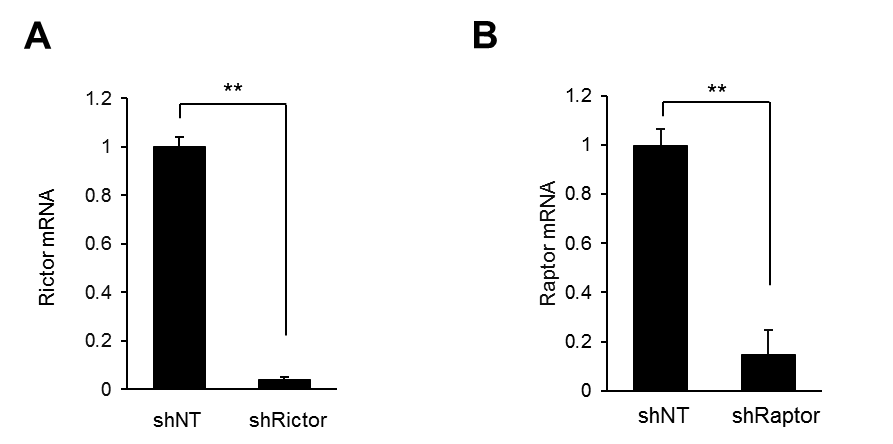
**

**Fig. S7**. Confirmation of Raptor and Rictor knockdown

The mRNA levels of Rictor (A) and Raptor (B) in EJ cells expressing either the non-targeting shRNA (shNC), Raptor shRNA (shRaptor) or Rictor shRNA (shRictor) were determined using real-time RT-PCR. ***P* <0.01 by Student’s *t*-test.

**Table S1.** Gene-specific primer sequences used for RT-PCR

| Gene name | Sequence | |
| --- | --- | --- |
| NOX1 | Forward | 5’-GTACAAATTCCAGTGTGCAGACCAC- 3’ |
|  | Reverse | 5’-CAGACTGGAATATCGGTGACAGCA -3’ |
| NOX2 | Forward | 5’-TCACTTCCTCCACCAAAACC- 3’ |
|  | Reverse | 5’-GGGATTGGGCATTCCTTTAT- 3’ |
| NOX3 | Forward | 5’-GGATCGGAGTCACTCCCTTCGCTG- 3’ |
|  | Reverse | 5’-ATGAACACCTCTGGGGTCAGCTGA- 3’ |
| NOX4 | Forward | 5’-CTCAGCGGAATCAATCAGCTGTG- 3’ |
|  | Reverse | 5’-AGAGGAACACGACAATCAGCCTTA- 3’ |
| DUOX1 | Forward | 5’-ATCAATCGGAACTCAAGTGTCTC- 3’ |
|  | Reverse | 5’-AACCAACACATGGTCCTCTCG- 3’ |
| DUOX2 | Forward | 5’-GCTACCATGTTCTTTCCGACG- 3’ |
|  | Reverse | 5’-GAGTGCGAGGAGCCATAGAT- 3’ |
| IL6 | Forward | 5’-ACTCACCTCTTCAGAACGAATTG - 3’ |
|  | Reverse | 5’-CCATCTTTGGAAGGTTCAGGTTG - 3’ |
| IL8 | Forward | 5’-ACTGAGAGTGATTGAGAGTGGAC - 3’ |
|  | Reverse | 5’-AACCCTCTGCACCCAGTTTTC- 3’ |
| Raptor | Forward | 5’-AATGCTGCAATCGCCTCTTCT - 3’ |
|  | Reverse | 5’-GCCAAAGGTAGGTTCCAGTCTG - 3’ |
| Rictor | Forward | 5’-TCCAAAGACTCGACAGTATGTGC - 3’ |
|  | Reverse | 5’- GGCTAGAAATCGTGCTTCTCTG- 3’ |
| Actin | Forward | 5’-CAAGAGATGGCCACGGCTGCT- 3’ |
|  | Reverse | 5’-TCCTTCTGCATCCTGTCGGCA- 3’ |
